# Nuclear ING3 Expression Is Correlated With a Good Prognosis of Breast Cancer

**DOI:** 10.3389/fonc.2020.589009

**Published:** 2021-01-05

**Authors:** Xiaoyan Wu, Chuang Chen, Bin Luo, Dandan Yan, Honglin Yan, Fangfang Chen, Feng Guan, Hao Wu, Jingping Yuan

**Affiliations:** ^1^Department of Pathology, Renmin Hospital of Wuhan University, Wuhan, China; ^2^Department of Breast and Thyroid Surgery, Renmin Hospital of Wuhan University, Wuhan, China

**Keywords:** breast cancer, immunohistochemistry, prognosis, disease-free survival, inhibitor of growth-3 expression

## Abstract

The inhibitor of growth (ING) family was discovered as the type II tumor suppressors, which regulated the proliferation, apoptosis, differentiation, angiogenesis, metastasis, and invasion of tumor cells through multiple pathways. ING3, a new member of ING family, has been reported to be downregulated in several types of tumors. However, few studies on ING3 in breast cancer have been reported. In this study, we investigated the expression of ING3 and determined its prognostic value in breast cancer. The immunohistochemistry was performed to evaluate the expression of ING3 in tissue microarrays (TMA) including breast cancer tissues (n=211) and normal breast tissues (n=50). In normal breast tissues, ING3 protein was detected in both the cytoplasm and nucleus. In breast cancer tissues, ING3 protein was principally detected in the cytoplasm. Compared with normal breast tissues, the expression of ING3 in nucleus was remarkably reduced in breast cancer tissues. The downregulated ING3 in nucleus was significantly correlated with clinicopathological characteristics including histological grade, lymph node metastasis, and the status of ER and PR. In HER2 positive-type and triple-negative breast cancer (TNBC) patients, it had the lower rate of nuclear ING3 with high expression than that in luminal-type. Moreover, Kaplan-Meier curves demonstrated that the reduced expression of ING3 in nucleus was correlated with a poorer 5-DFS and 5-OS of breast cancer patients. Importantly, multivariate Cox regression analysis suggested that the reduced expression of ING3 in nucleus was an independent prognostic factor in breast cancer. Our study comprehensively described the expression of ING3 in breast cancer for the first time and proved that it was an independent prognostic predictor of breast cancer, as well as a new idea for study of breast cancer.

## Introduction

Breast cancer, ranking second in cancer-related death among women worldwide, is the most common malignant tumor in women, which pose a serious threat to women’s physical and mental health (1). In the process of tumorigenesis, a variety of tumor suppressor genes mutate frequently, even though more targeted drugs are used in clinic, but most of them are achieved by suppressing overexpressed oncogenes. However, the treatment and recovery of lost tumor suppressor genes is still a key link (2).

Compared with most other tumors, the loss of tumor suppressor gene function plays a key role in the development of breast cancer (3). P53 is a tumor suppressor gene that has been widely studied at present. The inhibitor of growth (ING) family is the type II tumor suppressors, including ING1, ING2, ING3, ING4, and ING5, which participates in different stages of biological processes such as growth, proliferation, DNA repair, invasion, migration and apoptosis of tumor cells through a variety of mechanisms (4). ING3, a new member, is an important cofactor of p53, and it has become a research hotspot because of its structural differences from other family genes. ING3 is considered to be a tumor suppressor gene because of its biological functions such as inhibiting cell growth, regulating cell cycle arrest and inducing apoptosis in a p53-dependent manner (5). ING3 is usually expressed in normal human tissues, and its downregulation or deletion has been proved to be involved in the occurrence and development of a variety of tumors. Gunduz et al. found that the level of ING3 mRNA decreased in half of the primary head and neck squamous cell carcinoma, and the expression of ING3 decreased significantly in advanced and poorly differentiated tumors (6, 7). This suggests that the expression of ING3 is closely related to the staging and differentiation of the tumor. Yang H Y et al. reported that the low expression of ING3 protein is associated with tumor invasion of hepatocellular carcinoma (8). In addition, Wang et al. found that nuclear ING3 expression decreased while cytoplasmic ING3 expression increased in melanoma, and nuclear ING3 expression was negatively correlated with tumor size, depth of invasion, dedifferentiation, clinicopathological stage and poor prognosis (9). This suggests that the expression of ING3 in the nucleus can be used as a basis for evaluating and predicting the prognosis of patients with primary melanoma.

Although there is no related report of ING3 in breast cancer at this stage, ING4 and ING5, as members of the ING family, participate in the occurrence of breast cancer. Studies has shown that compared with benign epithelium, the nuclear expression of ING4 is decreased, and the cytoplasmic expression of ING4 is positively correlated with the expression of HER2 in breast cancer, which suggests that ING4 plays a role in the pathogenesis of breast cancer (10). The expression of ING4 was negatively correlated with histological grade of breast cancer. At the same time, the expression of ING4 is negatively correlated with the histological grade of breast cancer (11), and overexpressed ING4 can inhibit the formation of microvessel in tumor tissue to inhibit the occurrence and development of breast cancer and improve the disease-free survival rate (12). Some researchers have confirmed that the expression of ING5 in paracancerous tissues of breast cancer is significantly higher than that in cancerous tissues. And the expression of ING5 in primary tumor was higher than that in distant metastatic tumor (13). In addition, the overexpression of ING5 leads to decreased glucose metabolism, fat accumulation, autophagy and apoptosis in breast cancer cells (14). These finds suggested that the ING family might be closely related to breast cancer. While, the members of the ING family are highly homologous, whether the expression of ING3 relates to the prognosis of breast cancer is still elusive.

To investigate the role of ING3 in the development and prognosis of breast cancer, Tissue microarray (TMA) technology and immunohistochemistry were performed to evaluate ING3 expression in breast cancer. Furthermore, the correlation between the nuclear ING3 expression and clinicopathologic variables, the patient of 5-year disease-free survival (5-DFS) and 5-year overall survival (5-OS) were analyzed in breast cancer.

## Materials and Methods

### Tissue Samples

Formalin-fixed, paraffin-embedded tissues from breast cancer tissues (n=211) and normal breast tissues (n=50) were used for immunohistochemistry (IHC) analysis in this study. All specimens were randomly obtained from the 2002 to 2008 archives of the Department of Pathology, Renmin Hospital of Wuhan University. All samples of primary invasive breast cancer receiving no treatment prior to surgery. The most representative tumor areas were carefully selected to make tissue microarray (TMAs) by two professional breast pathologists. All clinicopathologic data were obtained from medical archives and were re-evaluated in the light of the latest pathological diagnostic criteria of WHO by two professional breast pathologists. Follow-up data were retrospectively obtained from all of the patients. All Patients were followed up for 5 years. The follow-up data of the postoperative outcomes were obtained from the first date of diagnosis to the date of metastasis/recurrence or death or last follow-up time. The use of human breast cancer was approved by the Ethics Committee of Renmin Hospital of Wuhan University.

### Immunohistochemical Staining

To analyze the expression of ING3 in breast cancer, TMA slides were used for immunohistochemistry (IHC) analysis by the Envision method in this study. ING3 antibody (1:1000, Proteintech, 10905-1-AP), Secondary antibody (DakoCytomation K0672), AEC/peroxidase (PO) (Maixin Biotechnology, AEC-0037). Two independent pathologists evaluated the expression of ING3 in nucleus and cytoplasmic by a double-blind manner. Considering the heterogeneity of immunohistochemical staining intensity and distribution, the evaluation of ING3 was scored by applying a semi-quantitative immunoreactivity scoring (IRS) system as described by Wang et al. (9). According to the staining intensities, the expression of ING3 in nucleus and cytoplasmic were categorized into four grades as follows: 0 (absence of staining, non-staining), 1 (weak staining, light yellow), 2 (moderate staining, brown yellow), and 3 (strong staining, dark brown). The percentage of ING3-positive cells in normal breast with nucleus and cytoplasmic and in breast cancer with nucleus were categorized into four grades as follows: 0 (0%), 1 (1%–33%), 2 (34%–66%), and 3 (67%–100%). According to the intensity and percentage scores, the final ING3 nuclear or cytoplasmic staining score was defined as follows: 0 to 3 as low expression, and 4 to 6 as high expression.

### Statistical Analysis

The SPSS 17.0 software was used to analyze data in this study. The χ^2^ test was applied to evaluate the differences of nuclear ING3 expression between breast cancer tissues and normal breast tissues. The Spearman’s rank correlation analysis was used to evaluate the correlation between the nuclear ING3 expression and the clinicopathologic variables, including age, histological grade, lymph node metastasis, TNM stage, the status of ER, PR and HER2, The Kaplan-Meier method and log-rank test were applied to evaluate the correlations between nuclear ING3 staining and patient 5-DFS and 5-OS. Cox regression model was applied for multivariate analysis. *P*<0.05 is considered significant.

## Results

### The Characteristics of the Tissue Samples

All tissue samples, breast cancer tissues (n=211) and normal breast tissues (n=50), were collected from the Department of Pathology, Renmin Hospital of Wuhan University. None of the breast cancer patients had received treatment, including chemotherapy, radiotherapy or adjuvant treatment, prior to surgery. The age of all breast cancer patients at the time of diagnosis ranged from 29 to 78 years old (median, 48.1 years). The main clinicopathologic variables in this study were shown in **Table 1**.

**Table 1:** The nuclear ING3 expression and clinicopathological parameters in breast cancer.

Clinicopathological parameters	Total Cases (n)	ING3 Staining in Nucleus		χ^2^	*P*
		Low expression	High expression		
Age (years)					
≤50	128	66	62	0.519	0.471
>50	83	47	36		
Histological grade					
1	36	13	23	14.794	0.001*
2	123	61	62		
3	52	39	13		
Lymph node metastasis					
Without	95	43	52	4.776	0.029*
With	116	70	46		
TNM stage					
I	13	5	8	3.281	0.194
II	135	69	66		
III	63	39	24		
Metastasis or recurrence in five years					
Without	132	63	69	4.813	0.028*
With	79	50	29		
Survival in five years					
Without	43	29	14	4.187	0.041*
With	168	84	84		
HER2					
Negative	160	80	80	3.362	0.067
Positive	51	33	18		
ER					
Negative	120	77	43	12.598	<0.001*
Positive	91	36	55		
PR					
Negative	117	70	47	4.157	0.041*
Positive	94	43	51		

Signifcant P-values are shown in italic type, *P < 0.05. TNM was evaluated in the light of the eight edition of AJCC. ER and PR were interpreted in the light of the 2010 ASCO/CAP guidelines. HER2 was interpreted in the light of the 2018 ASCO/CAP guideline.

### Nuclear ING3 Expression in Breast Cancer Tissues Is Lower Than That in Normal Breast Tissues

**Figure 1:**
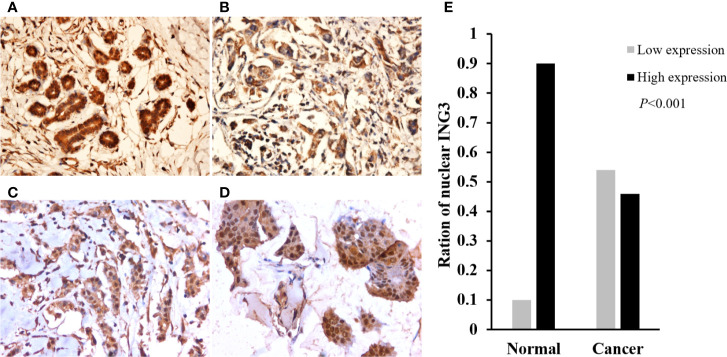
The expression and distribution of ING3 in representative tissue specimens. **(A)** The expression of ING3 in normal breast tissues. **(B)** Negative nuclear ING3 staining in breast cancer tissues. **(C)** Moderate nuclear ING3 staining in breast cancer tissues. **(D)** Strong nuclear ING3 staining in breast cancer tissues. **(A–D)** Magnification, ×200. **(E)** The summary of nuclear ING3 staining in breast cancer tissues and normal breast tissues. The intensity of nuclear ING3 expression in breast cancer tissues and normal breast tissues are indicated as follows: Low expression (light gray) and High expression (black). The expression of nuclear ING3 are significantly decreased in breast cancer tissues (*P* < 0.001).

To investigate the expression of ING3 in breast cancer, we performed the immunohistochemistry staining on TMA slides. In normal breast tissues, ING3 was located both in the cytoplasm and nucleus (**Figure 1A**). While, in breast cancer tissues, ING3 was principally located in the cytoplasm, and was rarely expressed both in the cytoplasm and nucleus (**Figures 1B–D**). The ration of the nuclear ING3 high expression was 46.4% (98/211) in breast cancer tissues, was 10% in normal breast tissues. The χ^2^ test showed that the expression of nuclear ING3 in breast cancer tissues was significantly lower than that in normal breast tissues (*P*<0.001) (**Figure 1E**).

### The Correlation Between the Nuclear ING3 Expression and the Clinicopathologic Characters in Breast Cancer

**Figure 2:**
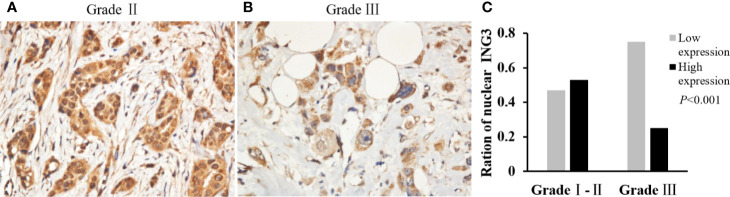
Analyses of nuclear ING3 expression in breast cancer with low-grade and high-grade. **(A, B)** The representative of nuclear ING3 staining in low-grade and high-grade breast cancer. **(A, B)** Magnification, ×400. **(C)** The χ^2^ test and Spearman’s rank result of nuclear ING3 staining in breast cancer with low-grade and high-grade (*P* < 0.001).

The χ^2^ test was performed to investigate the correlation between the nuclear ING3 expression and the clinicopathologic characters. As shown in **Figure 2**, in breast cancer tissues, the expression of nuclear ING3 in pathological grade III group (13/52) was lower than that in pathological grade I–II group (85/159) (χ^2 =^ 12.759, *P*<0.001) (**Figures 2A, B**), which suggested that the nuclear ING3 expression was negatively correlated with the histological grade (*P*<0.001) (**Figure 2C**).

**Figure 3:**
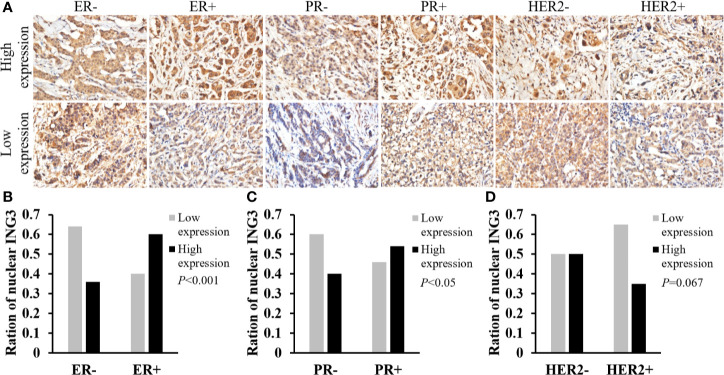
Analyses of nuclear ING3 expression in breast cancer with status of ER, PR and HER2. **(A)** The representative images of nuclear ING3 staining in different status of ER, PR and HER2. **(A)** Magnification, ×400. **(B–D)** The χ^2^ test and Spearman’s rank result of nuclear ING3 staining in breast cancer with in different status of ER, PR and HER2.

Then, we used the same method to study the correlation between the nuclear ING3 expression and the status of ER, PR, and HER2 in breast cancer tissues. The representative images of nuclear ING3 expression in different status of ER, PR and HER2 were shown in **Figure 3A**. The expression of nuclear ING3 was positively related to the status of ER and PR, that is the patients with ER or PR positive has a higher expression of nuclear ING3 (**Figures 3B, C**). By comparison, the expression of nuclear ING3 revealed no marked difference between HER2 positive and negative patients (*P*=0.067) (**Figure 3D**). In addition, we also found that the expression of nuclear ING3 was correlated with lymph node metastasis (*P*=0.029), but not with TNM stage and age (*P*=0.194 and *P*=0.471).

### The Difference Expression of Nuclear ING3 in Molecular Subtypes of Breast Cancer

**Figure 4:**
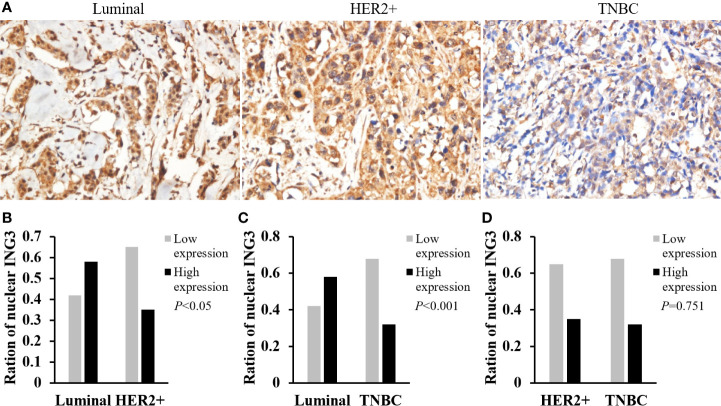
Analyses of nuclear ING3 expression in breast cancer with different molecular subtypes. **(A)** The representative images of nuclear ING3 staining in different molecular subtypes. **(A)** Magnification, ×400. **(B–D)** The χ^2^ test and Spearman’s rank result of nuclear ING3 staining in breast cancer with in different molecular subtypes.

According to the expression of ER, PR, and HER2, breast cancer can be divided into luminal-type (ER+, PR+, HER2-), HER2 positive-type (ER-, PR-, HER2+), and triple negative breast cancer (TNBC) (ER-, PR-, HER2-) (15). The representative images of nuclear ING3 expression in different molecular subtypes of breast cancer were shown in **Figure 4A**. The expression of nuclear ING3 in luminal-type patients was significantly higher than that in HER2 positive-type and TNBC patients (**Figures 4B, C**). At the same time, the nuclear ING3 expression showed no marked difference between HER2 positive-type and TNBC patients (**Figure 4D**). Thus, these results indicated that the low expression of ING3 in nucleus is correlated with more aggressive behavior of breast cancer, and ING3 may be one of the prognostic factors of breast cancer.

### High Expression of the Nuclear ING3 Is Associated With a Good Prognosis of Breast Cancer

**Figure 5:**
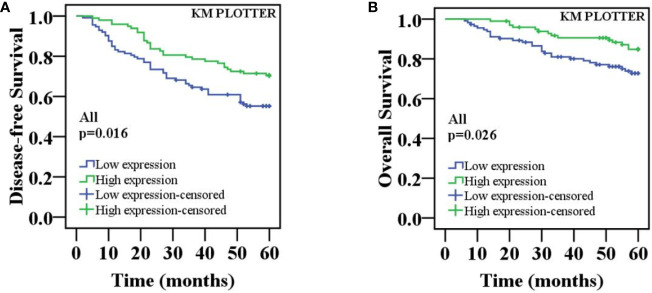
K-M survival curves for the correlation of the nuclear ING3 expression in breast cancer patients. **(A)** Patients with low expression of nuclear ING3 staining had a significantly worse 5-DFS than those with high expression of ING3 staining in 211 cases of breast cancer. **(B)** A similar conclusion for 5-OS.

To investigate whether the decreased nuclear ING3 staining serve as a prognostic biomarker in breast cancer, we followed all of the 211 patients and used the Kaplan-Meier method and log-rank test to analyze the follow-up data. The rate of 5-year disease-free survival (5-DFS) was 70.4% (69/98) in patients with high expression of nuclear ING3, while the 5-DFS rate was 55.8% (63/113) in those with low expression of nuclear ING3. The rate of 5-year overall survival (5-OS) was 85.7% (84/98) and 74.3% (84/113) in patients with high and low expression of nuclear ING3, respectively. Moreover, the K-M survival curves indicated that the nuclear ING3 expression was positively correlated with 5-DFS (*P*=0.016) and 5-OS (*P*=0.026) (**Figures 5A, B**). Collectively, these results indicated that the high expression of nuclear ING3 might be a good prognostic biomarker of breast cancer.

**Figure 6:**
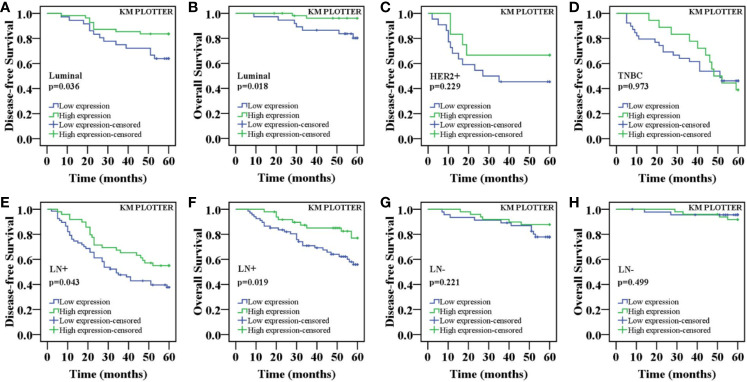
K-M survival curves for the correlation of the nuclear ING3 expression in breast cancer patients. **(A)** Patients with low expression of nuclear ING3 staining had a significantly worse 5-DFS than those with high expression of ING3 staining in luminal-type patients. **(B)** A similar conclusion for 5-OS in luminal-type patients. **(C, D)** K-M survival analysis of the nuclear ING3 expression was not statistically significant for 5-DFS in HER2 positive-type and TNBC patients. **(E–H)** K-M survival curves for the correlation between the nuclear ING3 expression and 5-DFS and 5-OS in breast cancer patients with or without lymph node metastasis. LN-, lymph node metastasis negative; LN+, lymph node metastasis positive.

The above results indicated that there were differences in the expression of nuclear ING3 in different molecular subtypes of breast cancer. Next, we explored whether the expression of nuclear ING3 affects the prognosis of different molecular subtypes of breast cancer. As displayed in **Figure 6**, the K-M survival curves indicated that the nuclear ING3 expression was positively correlated with 5-DFS (*P*=0.036) and 5-OS (P=0.018) in luminal-type patients (**Figures 6A, B**), but it could not well evaluate the prognosis of HER2 positive-type (*P*=0.229) and TNBC (*P*=0.973) patients (**Figures 6C, D**). In addition, we also found that nuclear ING3 could predict the prognosis of breast cancer patients in lymph node metastasis (LN+) group (**Figures 6E, F**) (*P*=0.043 and *P*=0.019), but not in lymph node negative (LN-) group (**Figures 6G, H**) (*P*=0.221 and *P*=0.499). These results suggested that nuclear ING3 played an important role in the prognosis of breast cancer.

To further verify the prognostic significance of nuclear ING3 in breast cancer, multivariate Cox regression analysis was performed. Related clinicopathologic variables were showed in **Tables 2**, **3**. The histological grade, lymph node metastasis, TNM stage and the status of HER2 had significant influences on 5-DFS and 5-OS. The status of ER and PR had no impact on 5-DFS and 5-OS. Meanwhile, the patients with high expression of nuclear ING3 had longer 5-DFS than those with low expression of nuclear ING3 (P=0.027; HR=1.771; 95% CI=1.066-2.994), but no similar conclusion was reached in 5-OS (P=0.129). These results indicated that the nuclear ING3 was an independent predictor for 5-DFS in breast cancer.

**Table 2:** Multivariate Cox regression analysis of prognostic variables including classical prognostic factors and nuclear ING3 for 5-year DFS in 211 cases of breast cancer patients.

Prognostic characteristics	B	HR	95% CI		*P*
			Lower	Upper	
Histological grade (1+2 vs 3)	-1.280	0.278	0.171	0.453	<0.001*
Lymph node metastasis (0 vs ≥1)	-1.044	0.352	0.181	0.683	0.002*
TNM stage (I+II vs III)	-0.582	0.559	0.319	0.980	0.042*
HER2 (negative vs positive)	-0.735	0.480	0.290	0.793	0.004*
ER (negative vs positive)	0.349	1.417	0.783	2.563	0.249
PR (negative vs positive)	-0.006	0.994	0.580	1.703	0.981
ING3 protein expression (low vs high)	0.572	1.771	1.066	2.944	0.027*

Signifcant P-values are shown in italic type, *P<0.05.

**Table 3:** Multivariate Cox regression analysis of prognostic variables including classical prognostic factors and nuclear ING3 for 5-year OS in 211 cases of breast cancer patients.

Prognostic characteristics	B	HR	95% CI		*P*
			Lower	Upper	
Histological grade (1+2 vs 3)	-1.565	0.209	0.106	0.411	<0.001*
Lymph node metastasis (0 vs ≥1)	-1.082	0.339	0.119	0.966	0.043*
TNM stage (I+II vs III)	-0.780	0.458	0.213	0.988	0.047*
HER2 (negative vs positive)	-1.009	0.364	0.191	0.695	0.002*
ER (negative vs positive)	0.762	2.144	0.922	4.985	0.077
PR (negative vs positive)	-0.167	0.846	0.422	1.699	0.639
ING3 protein expression (low vs high)	0.577	1.781	0.845	3.751	0.129

Signifcant P-values are shown in italic type, *P < 0.05.

## Discussion

ING3 is widely expressed in normal human tissues, such as heart, skeletal muscle, thymus, spleen, kidney, liver, pancreas, colon, ovary, testis, prostate, peripheral blood leukocytes, etc (16). In normal human tissues, ING3 is principally distributed in the cytoplasm, but it is occasionally observed in both the cytoplasm and nucleus, including tongue, esophagus, lung, skin, bladder, cervix and breast cells (17). In our study, ING3 was distributed both in the cytoplasm and nucleus in normal breast tissues, which was consistent with the report. Meanwhile, we also observed that, ING3 was mainly expressed in the cytoplasm in breast cancer tissues, and the expression of ING3 in the nucleus was significantly decreased. Similarly, the expression of ING3 decreased in many tumors, including human cutaneous melanoma (9), human head and neck cancers (7) and human primary hepatocellular carcinoma (8). Although the nuclear ING3 expression was significantly reduced in breast cancer tissues compared with normal breast tissues, it is not clear how the nuclear ING3 is reduced. Studies has shown that there might be a translocation of ING3 from nucleus to cytoplasm in melanoma (9). However, in our study, we did not notice that the significantly decrease of nuclear ING3 was accompanied by a remarkably increase in the expression of cytoplasmic ING3. One possible explanation for this discrepancy was that the nuclear to cytoplasm translocation of ING3 may be a partial reason for significantly reduce of nuclear ING3 in breast cancer tissues. The nuclear localization of ING3 protein was determined by the nuclear localization sequence (NLS) of ING3 gene (18). At the same time, Chen G et al. found that the decreased expression of ING3 in melanoma was degraded by ubiquitin-proteasome signal pathway (19). Therefore, the mechanism of the decreased expression of nuclear ING3 in breast cancer needs to be further studied.

In our study, we found that there was a correlation between the downregulation of ING3 in nucleus and clinicopathologic characters in breast cancer. The nuclear ING3 was negatively related with histological grade, which suggests that the decreased expression of ING3 in the nucleus was involved in the differentiation and played a continued role in the advancement and development of breast cancer. As the homologs of ING3, the degree of inhibition of ING4 protein expression was related to clinical stage, and the expression of ING4 in patients with lymph node metastasis was lower than that in patients without lymph node metastasis, indicating that ING4 participated in the occurrence and development of colon cancer (20). In breast cancer patients with the larger the tumor, the higher the stage, and the lower the expression of ING4 were more prone to lymph node metastasis (12). On the other hand, the nuclear ING3 was also negatively correlated with lymph node metastasis, which suggested that nuclear ING3 was related to the migration and metastasis of breast cancer. In agreement with our findings, Lu M et al. suggested that overexpression of ING3 might inhibit the migration and metastasis of hepatocellular carcinoma cells (21).

In addition, the nuclear ING3 was positively correlated with ER and PR status, which were closely related to endocrine therapy of breast cancer. Previous study had shown that low expression of ING4 reduced the efficacy of tamoxifen in breast cancer, by inhibiting ER activity in hormone-dependent breast cancer (22). As the homologous of ING4, ING3 might also be related to endocrine therapy of breast cancer. In this study, it had shown that there were no relationship between expression of ING3 in nucleus and age, TNM stage and HER2 status. Interestingly, the deletion of ING4 gene was associated with HER2 status in breast cancer (23). Meanwhile, the rate of high expression nuclear ING3 in breast cancer with luminal-type was higher than that with HER2-enriched and TNBC, which suggested that nuclear ING3 might play a role in distinguishing different subtypes of breast cancer.

All the above finding suggested that nuclear ING3 might play a key role, at least in part, in predicting the prognosis of breast cancer. Prognostic molecular biomarkers are valuable for evaluating the survival status of patients and assisting tumor control. It had been well demonstrated that ING3 might be a positive independent factor in melanoma, human primary hepatocellular carcinoma and head and neck cancer (6, 8, 9). Similarly, our survival analysis also showed that in luminal-type breast cancer and lymph node metastasis group, the lower nuclear ING3 expression, the poorer 5-DFS and 5-OS. Moreover, the independent prognostic biomarker of nuclear ING3 in breast cancer patients was revealed based on the multivariate Cox regression analysis.

In conclusion, our study showed that the expression of nuclear ING3 was significantly decreased in breast cancer, which was closely related to the clinicopathological parameters and might be used as an independent prognostic factor to evaluate the 5-DFS breast cancer patients. This study implemented a comprehensive analysis of the expression of ING3 in breast tissue for the first time, which provided a new idea and direction for better comprehensive understanding of breast cancer.

## Data Availability Statement

The raw data supporting the conclusions of this article will be made available by the authors, without undue reservation.

## Ethics Statement

The studies involving human participants were reviewed and approved by Renmin Hospital of Wuhan University (WDRY2019-K010). Written informed consent for participation was not required for this study in accordance with the national legislation and the institutional requirements. Written informed consent was not obtained from the individual(s) for the publication of any potentially identifiable images or data included in this article.

## Author Contributions

JY designed and supervised the study. XW performed experiments and data analysis, drafted the manuscript, and prepared the figures. CC and BL collected the data of patients and followed up. FG and FC evaluated the immunohistochemical staining of all sections. DY, HY, and HW provided technical assistance with the data analysis. DY revised the manuscript. All authors contributed to the article and approved the submitted version.

## Funding

This work was supported by grants from Science and Technology Planning Project of Wuhan (Grant No. 2017060201010172) and Guidance Foundation of Renmin Hospital of Wuhan University (Grant No. RMYD2018M27).

## Conflict of Interest

The authors declare that the research was conducted in the absence of any commercial or financial relationships that could be construed as a potential conflict of interest.

## References

[1] Yu BH Li BZ Zhou XY Shi DR Yang WT Cytoplasmic FOXP1 expression is correlated with ER and calpain II expression and predicts a poor outcome in breast cancer. Diagn Pathol (2018) 13(1):36. 10.1186/s13000-018-0715-y 29848352 PMC5977746

[2] Birkeland AC Ludwig ML Spector ME Brenner JC The potential for tumor suppressor gene therapy in head and neck cancer. Discov Med (2016) 21(113):41–7. PMC4772772 26896601

[3] Sanchez-Tapia C Wan FY Fastest time to cancer by loss of tumor suppressor genes. Bull Math Biol (2014) 76(11):2737–84. 10.1007/s11538-014-0027-7 PMC4282520 25338553

[4] Hu GW Yan XW Qin YJ Nie HT Molecular cloning and expression analysis of inhibitor of growth protein 3 (ING3) in the Manila clam, Ruditapes philippinarum. Mol Biol Rep (2014) 41(6):3583–90. 10.1007/s11033-014-3221-7 24566680

[5] Gou WF Sun HZ Zhao S Niu ZF Mao XY Takano Y Downregulated inhibitor of growth 3 (ING3) expression during colorectal carcinogenesis. Indian J Med Res (2014) 139(4):561–7. PMC4078494 24927342

[6] Gunduz M Beder LB Gunduz E Nagatsuka H Fukushima K Pehlivan D Downregulation of ING3 mRNA expression predicts poor prognosis in head and neck cancer. Cancer Sci (2008) 99(3):531–8. 10.1111/j.1349-7006.2007.00708.x PMC11159728 18081876

[7] Gunduz M Ouchida M Fukushima K Ito S Jitsumori Y Nakashima T Allelic loss and reduced expression of the ING3, a candidate tumor suppressor gene at 7q31, in human head and neck cancers. Oncogene (2002) 21(28):4462–70. 10.1038/sj.onc.1205540 12080476

[8] Yang HY Liu HL Tian LT Song RP Song X Yin DL Expression and prognostic value of ING3 in human primary hepatocellular carcinoma. Exp Biol Med (2012) 237(4):352–61. 10.1258/ebm.2011.011346 22550337

[9] Wang Y Dai DL Martinka M Li G Prognostic significance of nuclear ING3 expression in human cutaneous melanoma. Clin Cancer Res (2007) 13(14):4111–6. 10.1158/1078-0432.CCR-07-0408 17634537

[10] Jiang L Zhang X Xiang C Geradts J Wei Q Liang Y Differential cellular localization of CELSR2 and ING4 and correlations with hormone receptor status in breast cancer. Histol Histopathol (2018) 33(8):835–42. 10.14670/HH-11-979 29489009

[11] Zhang L Wang Y Zhang F Wang Y Zhang Q Correlation between tumor suppressor inhibitor of growth family member 4 expression and microvessel density in breast cancer. Hum Pathol (2012) 43(10):1611–7. 10.1016/j.humpath.2011.11.018 22436625

[12] Byron SA Min E Thal TS Hostetter G Watanabe AT Azorsa DO Negative regulation of NF-kappaB by the ING4 tumor suppressor in breast cancer. PLoS One (2012) 7(10):e46823. 10.1371/journal.pone.0046823 23056468 PMC3464231

[13] Song Y Zhao S Qi F Zheng H Nucleocytoplasmic translocation of ING5 protein in breast cancer and its correlation with poor clinicopathological characteristics of breast cancer. Xi bao yu fen zi mian yi xue za zhi = Chin J Cell Mol Immunol (2018) 34(1):53–8. 29595458

[14] Ding XQ Zhao S Yang L Zhao X Zhao GF Zhao SP The nucleocytoplasmic translocation and up-regulation of ING5 protein in breast cancer: a potential target for gene therapy. Oncotarget (2017) 8(47):81953–66. 10.18632/oncotarget.17918 PMC5669862 29137236

[15] Makki J Diversity of Breast Carcinoma: Histological Subtypes and Clinical Relevance. Clin Med Insights Pathol (2015) 8:23–31. 10.4137/CPath.S31563 26740749 PMC4689326

[16] Nagashima M Shiseki M Pedeux RM Okamura S Kitahama-Shiseki M Miura K A novel PHD-finger motif protein, p47ING3, modulates p53-mediated transcription, cell cycle control, and apoptosis. Oncogene (2003) 22(3):343–50. 10.1038/sj.onc.1206115 12545155

[17] Gou WF Yang XF Shen DF Zhao S Sun HZ Luo JS Immunohistochemical profile of ING3 protein in normal and cancerous tissues. Oncol Lett (2017) 13(3):1631–6. 10.3892/ol.2017.5632 PMC5403501 28454301

[18] Soliman MA Riabowol K After a decade of study-ING, a PHD for a versatile family of proteins. Trends Biochem Sci (2007) 32(11):509–19. 10.1016/j.tibs.2007.08.006 17949986

[19] Chen G Wang Y Garate M Zhou J Li G The tumor suppressor ING3 is degraded by SCF(Skp2)-mediated ubiquitin-proteasome system. Oncogene (2010) 29(10):1498–508. 10.1038/onc.2009.424 19935701

[20] You Q Wang XS Fu SB Jin XM Downregulated expression of inhibitor of growth 4 (ING4) in advanced colorectal cancers: a non-randomized experimental study. Pathol Oncol Res POR (2011) 17(3):473–7. 10.1007/s12253-010-9301-7 21626442

[21] Lu M Chen F Wang Q Wang K Pan Q Zhang X Downregulation of inhibitor of growth 3 is correlated with tumorigenesis and progression of hepatocellular carcinoma. Oncol Lett (2012) 4(1):47–52. 10.3892/ol.2012.685 22807958 PMC3398365

[22] Keenen MM Kim S Tumor suppressor ING4 inhibits estrogen receptor activity in breast cancer cells. Breast Cancer (2016) 8:211–21. 10.2147/BCTT.S119691 PMC5117803 27895513

[23] Tapia C Zlobec I Schneider S Kilic E Guth U Bubendorf L Deletion of the inhibitor of growth 4 (ING4) tumor suppressor gene is prevalent in human epidermal growth factor 2 (HER2)-positive breast cancer. Hum Pathol (2011) 42(7):983–90. 10.1016/j.humpath.2010.10.012 PMC3103605 21315418

[24] Wu X Chen C Luo B Yan D Yan H Chen F Nuclear ING3 Expression is Correlated With a Good Prognosis of Breast Cancer. Res Square Preprints (2020). 10.21203/rs.3.rs-33232/v1 PMC7813678 33469513

